# Odontological Society of Great Britain

**Published:** 1876-10

**Authors:** 


### ARTICLE V.

### *Odontological Society of Great Britain.*

Monthly Meeting, June 12th, 1876.

Charles Vasey, Esq., President, in the Chair. The President announced that Mr. Moon had presented the Library with two volumes of Mr. Bryant's "Practice of Surgery." He regretted to state that, owing to extreme pressure of business, Dr. Murie would be unable to be present to read his paper, but it would be read some time during the winter. He hoped the evening would be filled up by the hearing and discussion of casual communications.

Mr. Weiss called attention to a case of deposition of tartar on the buccal side of a block. In extent it was about $1\frac{1}{4}$ inch in length, and $\frac{5}{8}$ths of an inch in thickness. What made it somewhat interesting and remarkable was that there were clear indications on the grinding surface of the block that this side of the mouth had been freely used for mastication; and, what made it still further remarkable was that such a piece of foreign body in the mouth could cause disfigurement of the face, causing the cheek to be thrust out without the patient being aware of it. He also called attention to a new flask which he had constructed, which had been vulcanised over 1,400 times, and had been in constant use for seven years. It had a shaft and a rod on each side; and the upper part went down readily, steadily, and perfectly perpendicularly, which prevented the teeth being broken by the closing of the flask, as was the case in many other flasks, arising from the fact that the two halves did not go into the true position. He thought that the contrivance was a friend that could always be relied upon for doing its work.

Dr. Sanger said that the case mentioned by Mr. Weiss reminded him of one, many years ago, of a woman, 60 years of age, who was subject to rheumatic gout, and who had concretions, considerably larger than those mentioned, all round the teeth of the lower jaw. He fancied that the rheumatic gout had something to do with the formation of the concretions. He also remembered seeing a formation of the same kind in the sub-lingual gland.

The President mentioned the case of a woman who had a large mass formed in her mouth, the same size and the same description which formed over the molar tooth, and she came to the hospital to have the *exostosis* treated.

Mr. Turner, referring to the case mentioned by Mr. Weiss, said it was to him remarkable to see such an accumulation of tartar in the part of the mouth which had been freely used for mastication, and it showed how much might be done by gradually increased pressure. It was also strange to see how the tartar, which was deposited in a soft state, could make room for itself. The Society was much indebted to Mr. Weiss for calling attention to the flask which he had constructed, but he (Mr. Turner) thought that the flask which enables the operator to hold the teeth and the model in the same position, before he commenced to pack in the rubber, would be most valuable, and that was the kind of flask that had been invented by Mr. Brunton, who had before the profession for two years what is known as the Contour flask.

Mr. Stocken mentioned a case in which a concretion, passed through the sub-lingual gland every third year. It was about half an inch in length and $\frac{3}{4}$ths of an inch in width.

Mr. Lyons said he had a case of a lady, 22 years of age, where the four front permanent incisors were loose, and the whole of the alveolus disappeared. There was nothing remarkable in the disease, except that it occurred at such an early age.

Mr. Sewill thought that there might be some relation between the deposition of tartar and rheumatic gout diathesis, but it could hardly be that indicated by Dr. Sanger. He thought that gouty concretions of any kind were not analagous to salivary deposit. The tartar was more akin to the concretions of the bladder which fastened round a foreign body. So far as he knew, in the mouth there was no special concretion of tartar from the glands, and therefore it was not in any direct relation to those chalky stones which were found in gout. Tartar, besides being made of earthly matter, contained foreign particles, such as epithelium scales, and other matter found in the mouth.

Mr. Lyons, in answer to the President, said that the teeth in the young lady he had referred to had been gradually loosening. The disease had been about three years in duration.

In answer to Mr. Turner, Mr. Lyons stated that the teeth had to be removed because they were so very loose.

Mr. Canton: Hearing that the paper for this evening was put off, and having two cases which have interested me, I have hurriedly put the notes down in order to bring them before you this evening, therefore if they are not as perfect as they might be, perhaps you will kindly excuse it. The history of the first case is as follows—A young lady, at the age of 21, had a severe fall during an attack of scarlatina. One front tooth she distinctly states was loose before she had the fall, but four years after this two other front teeth had become so loose that the three were extracted and put on to a gold plate at the age of 25 years. Last July (six years since the upper front teeth were extracted) the four lower incisors had to be extracted on account of the extreme looseness, and also the right lower first molar, on account of the decay of the posterior fang apparently from pressure, caused by the second molar. This tooth was very loose as well. This brings the lady to the age of 31 years, and wearing three upper and five lower artificial teeth. I may mention that she is the daughter of a medical man, and one of a very large family, that no other members suffer in the same way, and that her father can in no way account for it, as she has always been a very healthy person, and had no illness whatever, besides the scarlatina mentioned. Her gums are, to all appearance, perfectly healthy, but rapidly retreating from the teeth, the fangs of nearly every tooth are quite exposed, and all the teeth more or less loose. I saw this patient on the 17th of May when she came to me on account of the left upper first bicuspid having dropped out, and which I added to the plate she already wears. I have brought with me the four lower incisors which I will pass round. They are to all appearances perfectly healthy, and every other tooth is so also, except the molar extracted which I think is accounted for by pressure. The second case, which is very similar, is as follows, and I will read it as written for me by the patient:—"When first I noticed my teeth getting loose it was about the year 1870. Three years after that I went to a dentist who scraped them. I tried several times to fasten them, but all to no purpose. My gums used to be so very full and a dark red, also to swell sometimes. I waa subject to gumboils. For years I noticed a thick matter on the teeth. About two years ago this discharge from the gums became greater but not so thick; now it is all about my mouth in the morning; before, it was, confined to the teeth only. I never had a serious illness in my life, and have always had good health with the exception of colds, headaches, and a very low nervous feeling; my age is 26. I cannot in any way account for my teeth becoming loose." I saw this patient for the first time on August 9th last year, and then extracted the two upper central and one lateral incisors, which were perfectly loose, and every other tooth in her head was in a similar condition to the first case—namely, her gums very much receded, roots all exposed, and teeth all more or less loose, especially one lower incisor. I saw no sign of any discharge from the gums, which appeared quite healthy. I could get no history of a fall or blow or illness, or in fact anything to account for the state of her mouth. I have the teeth extracted with me, and will pass them round. These two cases I have brought before this Society as I think there is a great similarity between them, and because I should very much like to hear the individual opinions of the members. They are the only two cases I have ever met with, or even heard of, in which the teeth have begun to loosen and drop out perfectly sound between the age of twenty and twenty-one. In the first case there is the history of a fall, which possibly may have given rise to the loosening; but I should have expected to find some discoloration in the teeth, which is generally the result of a blow or fall as well as the loosening, but this is not so in either of these cases; the teeth are of a perfectly good color, and also after a blow or fall one would hardly expect every tooth in the head to loosen.

Mr. Sewell mentioned a case he had watched for seven or eight years of complete absorption of the root of a permanent tooth. It became gradually looser until it was removed by the finger nail; but there was a history of injury which had occurred many years previously. He had several patients, young ladies, about the age mentioned by Mr. Canton, under his care at present, and they were losing their teeth in the way that had been described, the alveolus being slowly absorbed. The teeth were sometimes so loose that they had to be removed. In some of these cases the general health might be suspected; but in most of them there was very little history to account for the local disease. The treatment he adopted was to make the best model he could, using liquid plaster of Paris, and making a vulcanite frame all round the mouth, and to protect the loose teeth from the bite and from change of temperature, the frame embracing accurately the necks of the teeth.

Mr. Hutchinson said that three months ago he had a case similar to the one related by Mr. Canton, in which without any apparent cause, loosening of the whole of the teeth in the upper and lower jaws occurred. From experience he was quite convinced that, in a tooth the root of which became suddenly conical, loosening was much more likely to occur than when the roots were of the round shape.

Mr. Oakley Coles mentioned a case he had of a girl 23 years of age, a worker in artificial wax flowers. Her gums were very much inflamed at the margins, and the teeth were loose and he thought very wide at the cutting edge. The tartar was of a dark colour, exceedingly hard, and passed at some distance from the neck of the teeth towards the alveolar cavity. He removed as much of the tartar as he possibly could, then scarified the gums afterwards, applying to them chlorate of potash ground up in glycerine. None of the teeth were lost, and the girl got better. He could not say whether the nature of her occupation had anything to do with the condition of the mouth. There was no history of syphilis, and the girl was strong in every respect.

Mr. Henry, referring to the remarks of Mr. Sewill, thought that the glands themselves were great contributors to the amount of tartar deposits.

Mr. Sewill said that what he wished to state was that the tartar was derived from the saliva as the saliva was derived from the glands.

Mr. C. S. Tomes exhibited some models sent by Dr. Moffatt, of Edinburg, which were interesting as showing how a very unpromising-looking mouth could be brought into a tolerably satisfactory form by the extraction of the teeth, and by the patient learning to put a pretty constant pressure on one of the teeth with the finger. He also called attention to some peculiarities he had observed in the development and succession of the teeth and poison fangs of vipers.

The President called attention to a model of a cleft palate in which the incisor tooth was not only turned round but bent upwards to a horizontal position, sticking out in a very awkward and frightful-looking manner. Such cases were generally treated at a very early age, but the woman in this case was over thirty, and the hare lip had not been operated on. The supernumerary tooth and the lateral incisor were on the other side of the gap from what it would have been if the gap depended upon non-union of the intermaxillary bone with the two maxillary bones. The division generally seemed to be between the lateral and central incisors.

Mr. Sewill related a case of a young lady, fourteen years of age, whose teeth were some months ago broken into fragments by a blow from a stone. It was necessary to extract the front teeth, and as the mouth was badly adapted for artificial teeth he thought it better to close the gap in front by pressing the teeth together and pressing them in before asking the patient to wear artificial teeth. A plate was prepared and pressed in the teeth, and after wearing it for some time there was very little deformity in the front of the mouth. If the plate had been constantly worn there would have been greater improvement, but, as it was, he had no doubt that the teeth would continue to improve from the natural growth of the jaw.

Mr. Oakley Coles said the President's model of congenital cleft palate showed negatively the influence of the operation of hare-lip performed in early life as compared with the relative position of the jaws at an adult age. He had never before seen a model of the mouth of a patient thirty years of age in which there was anything approaching perfect articulation in the upper an l lower teeth. In many cases it might be impossible to postpone to a late period the operation for hare-lip, but where it could be done it would be very desirable, as it would preserve the relative position of the upper and lower jaw much more perfectly than if the operation was done earlier in life, and would make a much more symmetrical arch for operation or for the application of an artificial palate later on in life.

The President was afraid that deferring the operation oı uniting the lips became so serious in the matter of articulation that very few in the present day would consent to its being deferred. The difficulty, too, in feeding a young child made it very desirable to have the lip united as early as possible.

After some questions put by Mr. Gaddes and Mr. Coleman had been answered by Mr. Tomes in reference to the communication he had made, the meeting, which was the last of the session, was adjourned.—*Monthly Review of Dental Surgery.*

---

